# The virtual and the physical: two frames of mind

**DOI:** 10.1016/j.isci.2020.101965

**Published:** 2020-12-17

**Authors:** Bilge Mutlu

**Affiliations:** 1 Department of Computer Sciences, University of Wisconsin–Madison, Madison, WI 53706, USA

**Keywords:** Cognitive Neuroscience, Artificial Intelligence, Social Sciences, Psychology

## Abstract

Virtual and physical embodiments of interactive artificial agents utilize similar core technologies for perception, planning, and interaction and engage with people in similar ways. Thus, designers have typically considered these embodiments to be broadly interchangeable, and the choice of embodiment primarily depends on the practical demands of an application. This paper makes the case that virtual and physical embodiments elicit fundamentally different “frames of mind” in the users of the technology and follow different metaphors for interaction, resulting in diverging expectations, forms of engagement, and eventually interaction outcomes. It illustrates these differences through the lens of five key mechanisms: “situativity, interactivity, agency, proxemics, and believability”. It also outlines the design implications of the two frames of mind, arguing for different domains of interaction serving as appropriate context for virtual and physical embodiments.

## Graphical abstract

**Figure:**
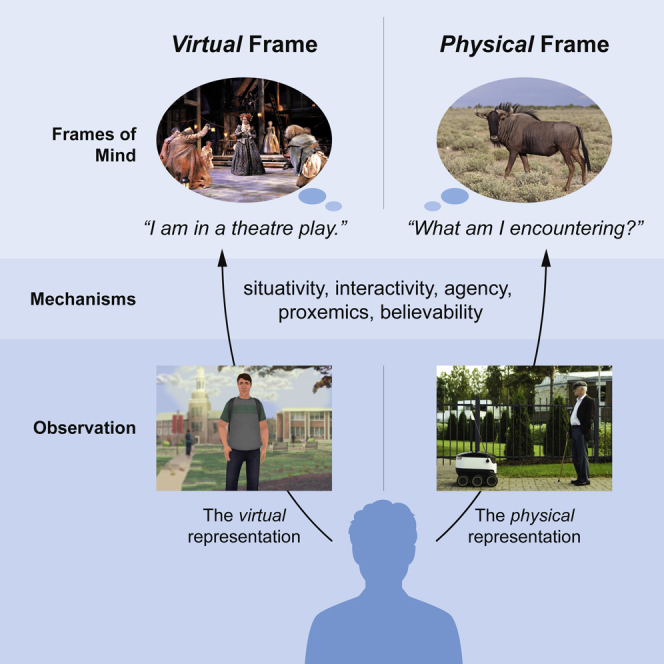

## Introduction

Since their inception, research communities concerned with the design of interactive “machines,” or computer systems, including virtual characters and social robots, have grappled with the “embodiment question”: What are the effects of having a “body”? What are differences between different forms of embodiment? Are some forms of embodiment “better,” i.e., more effective, natural, and intuitive in facilitating interaction with people, than others? If so, why? Early work in these communities has emphasized the importance of interactive systems to have a body over disembodied systems, such as speech-based interfaces (e.g., Cassell, 2001), explored how such embodiments must be designed (e.g., Gockley et al., 2005), and studied how different forms of embodiment affect human-machine interaction, making comparisons most commonly among disembodied (conversational agents), virtually embodied (virtual characters), and physically embodied (social robots) systems (e.g., Kiesler et al., 2008). For a comprehensive characterization of different forms of embodiment and further discussion of the embodiment question, see Li (2015) and Deng et al., 2019.

But what is “embodiment”? Cassell (2001) argued that the design of a computational system reflects a set of representational choices, e.g., how the capabilities of the systems are represented in its user interface and that representations that follow a human model are particularly effective at facilitating interaction with the system. And to effectively follow a human model, these representations must include a discernible and familiar body, that is, they must be “embodied”. In this paper, the term “representation” refers to the collection of representational choices for a system and is used to discuss the abstract concept of a system's representation, while the term “embodiment” refers to specific choices in how the system is designed to represent its capabilities to its user.

In the last decade, a large body of literature has emerged with the aim of better characterizing the different forms of embodiment used in the design of artificial agents and gaining a better understanding of the differences in how people respond to and perceive them. This literature has drawn a contrast between “virtual embodiments”, including disembodied systems, such as voice assistants and graphical or virtual embodiments, and “physical embodiments”, including physical or hybrid embodiments, such as social robots. Meta-analyses of this body of work indicate that virtual and physical embodiments differ in patterned ways: in general, physical embodiments outperform virtual ones in measures of the extent to which people favor them and how well they support user task and goals Deng et al., 2019, and physical, collocated presence is the primary discriminating factor for these embodiments Li (2015). Despite this general pattern, our understanding of the embodiment question is far from being complete; the findings of some studies diverge from this pattern, as illustrated by the case study presented in this section, and we lack an understanding of the underlying causes of such differences. This paper aims to close this gap by proposing a model for why human responses to and perceptions of virtual and physical embodiments markedly differ, toward generating testable theory that can facilitate future research on the “embodiment question”.

### A case study in differences

The body of literature on the embodiment question suggests that, in general, physical embodiments outperform virtual embodiments in how well they support desirable “interaction outcomes”, such as user task performance, experience, and perceptions of the system. In other words, these findings suggest that, in the context of the embodiment question, physical embodiments are “better” than virtual embodiments. However, the literature also includes a number of examples where this conclusion does not predict differences elicited by physical and virtual embodiments. Although one might argue that these instances are outliers or exceptions, another possibility is that directly comparing physical and virtual embodiments to measure the differences in the “magnitude” of their effects on interaction outcomes is not an appropriate way to understand the underlying reason for such differences or lack thereof. To gain such understanding, research must go beyond the black box treatment of the embodiment effect phenomenon and identify “mechanistic” differences in how embodiment affects people by determining the underlying causes, factors, and organization of this phenomenon Craver and Tabery (2019).

**Figure 1:**
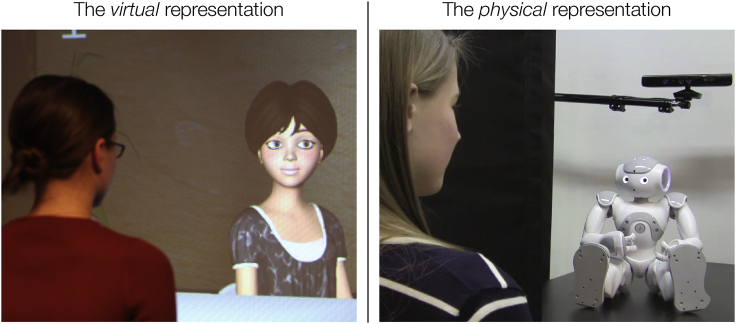
Experimenters demonstrating interaction with the representations used in the case study The virtual character Andrist et al. (2013) on the left and the social robot on the right Andrist et al. (2014). Copyright information: left: Springer; right: Bilge Mutlu.

To illustrate how the research question can be reformulated, from “which embodiment is better” to “how do different forms of embodiment affect people differently,” this paper will consider a case study. In a two-part study, Andrist et al. (2013, 2014) investigated how agents and robots should establish and maintain eye contact with their users. The representations used in the study are shown in Figure 1. The literature on human gaze suggests that increased eye contact increases the favorability of personality characteristics Brooks et al. (1986), although the literature also suggests that an inordinate amount of eye contact, e.g., staring, can result in discomfort and an escape response Ellsworth et al. (1972). These findings suggest that there is an optimal amount of eye contact among interlocutors and that they must break eye contact by averting their gaze to avoid staring. Because the literature on human gaze does not offer an adequate model of “gaze aversion”, Andrist et al. (2013, 2014) analyzed video data of 24 human dyads interacting in an interview scenario, developed a model that characterized three forms of gaze aversions, and implemented this model on a virtual agent (study I, reported in Andrist et al. (2013)) and a social robot (study II, reported in Andrist et al. (2014)).

The studies both followed a within-participants design, manipulating the gaze aversion behaviors of the virtual agent or the robot: (1) no gaze aversion, (2) gaze aversion timed to be incongruent with the model, and (3) gaze aversion timed to be congruent with the model. In the studies, participants performed a set of conversational tasks that involved “interviewing” a virtual character or a social robot that autonomously conversed with them for a job at the university's library. The tasks included (1) intent, (2) floor management, (3) attributions of thoughtfulness, and (4) disclosure. The first two tasks were designed to facilitate an understanding of how gaze aversion facilitated conversational mechanisms, specifically, to determine whether or not correctly timed gaze aversions improve interaction flow. The third task tested whether “cognitive” gaze aversions, when speakers look up before answering a question, improved attributions of thoughtfulness to the robot based on its responses. Finally, the fourth task investigated the role of gaze aversion in intimacy regulation, specifically whether or not listener gaze aversion elicits more disclosure from speakers. Hypotheses associated with the tasks predicted that the agent/robot, (1) would be perceived as more purposeful in task 1, intent, (2) would hold the floor longer in task 2, floor management, (3) would appear more thoughtful in task 3, attributions of thoughtfulness, and (4) would elicit more disclosure in task 4, disclosure, when it averted gaze congruently with the model than when it averted its gaze incongruently or when it did not avert its gaze.

The findings from the two studies, summarized in Table 1, showed that, across the two studies, gaze aversion worked as expected in supporting conversational mechanisms: there were fewer interruptions in question-answer sequences and in conversational turn-taking. On the other hand, the two studies differed in the effects of gaze aversion on perceptions of thoughtfulness and on the amount of disclosure: participants found the robot to be more thoughtful but not with the virtual agent, and they disclosed to the agent more but not to the robot, when it averted its gaze at times that were dictated by the model. These differential results are not consistent with the predictions of the body of work on the embodiment question—that physical embodiments would elicit stronger interaction outcomes than virtual embodiments. These results suggest not differences in the magnitude of expected effects but differences in the functioning of specific cognitive mechanisms that system embodiment affects.

**Table 1:** Summary of findings from the two studies

Task	Hypothesis	Agent	Robot
Intent	The agent would be perceived as more purposeful	Supported	Supported
Floor management	The agent would hold the floor longer	Supported	Supported
Attributions of thoughtfulness	The agent would appear more thoughtful	Not supported	Supported
Disclosure	The agent would elicit more disclosure	Supported	Not supported

Worthy of note is that these differences could result from errors in experimental implementation, sampling, or measurement or other alternative explanations might exist. While acknowledging these possibilities, this paper will argue that these differences stem from “experiential” differences between virtual and physical representations, as discussed in the paragraphs below.

## A working hypothesis

**Figure 2:**
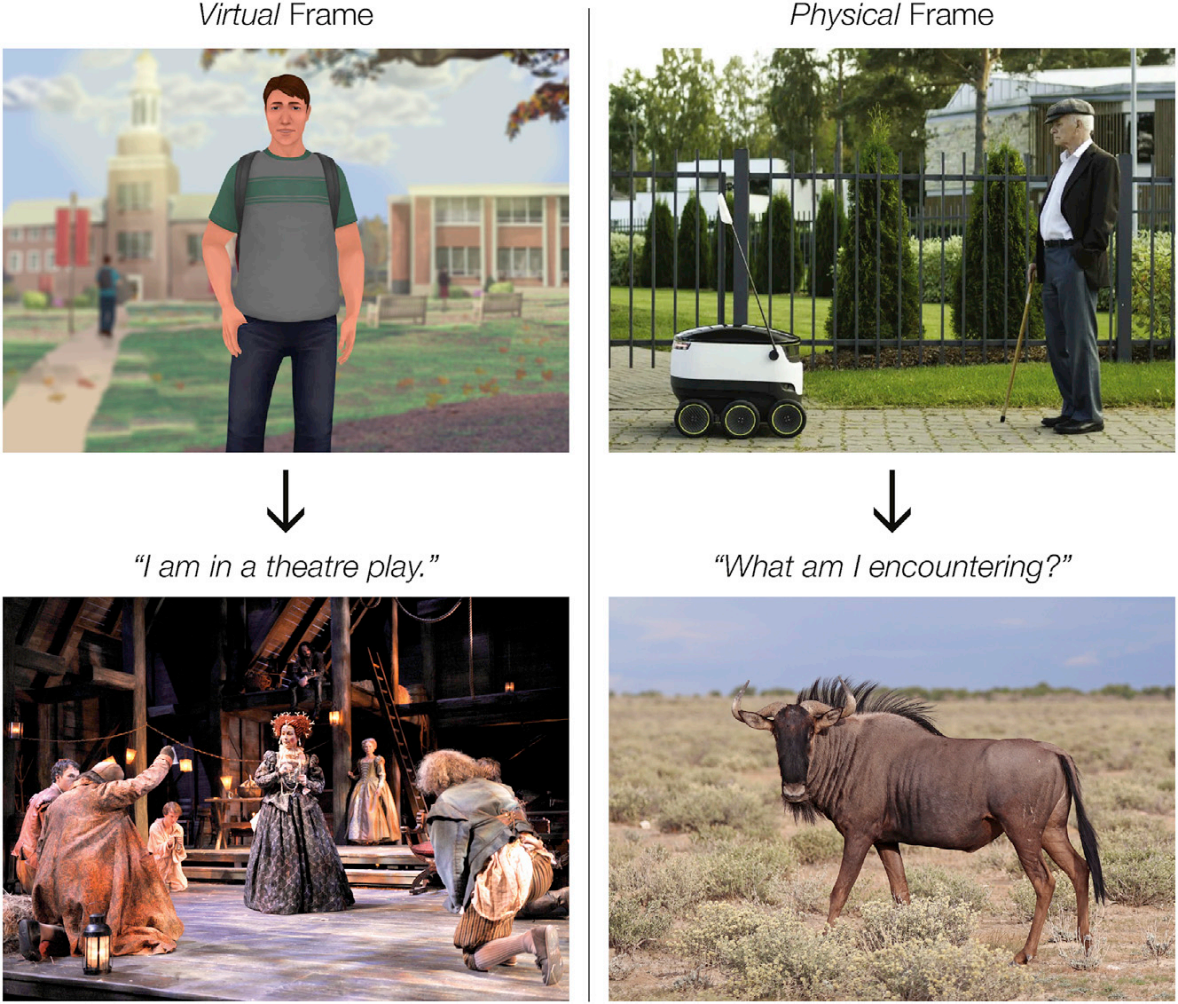
The working hypothesis of this paper is that virtual and physical representations elicit fundamentally different frames of mind Virtual representations eliciting the mindset, “I am in a theater play,” and physical representations eliciting the mindset, “What am I encountering?” Copyright information: top left: Virtual human, courtesy of Kognito. www.kognito.com; top right: Starship Technologies; bottom left: Chicago Shakespeare Theater's 2011 production of *Elizabeth Rex*. Photo by Liz Lauren; bottom right: Charles James Sharp, CC BY-SA 4.0, via Wikimedia Commons.

This paper proposes a hypothesis that will serve as one explanation of “why” virtual and physical representations differ “experientially” beyond their differences in material, form, and technology. This hypothesis posits that virtual and physical embodiments are not two faces of the same coin or mere representational choices that mechanistically work the same way but instead “they elicit two discrete and distinct frames of mind in people who interact with and experience them”, as illustrated in Figure 2. In brief terms, “virtual and physical representations elicit alternative frames of mind”.

“Frames of mind,” or “cognitive frames,” refer to mental templates that individuals apply to information, entities, or environments that they encounter to make sense of them and to determine appropriate courses of action Walsh (1995). In the context of this discussion, individuals adopt different frames of mind when they encounter virtual and physical embodiments and apply different schemas in making sense of what they are encountering and determining how to interact with them, which results in different expectations, perceptions, behavior, and eventually interaction outcomes.

To illustrate these frames of mind, consider two scenarios, one involving a virtual representation and the other involving a physical representation. Note that the goal of providing these scenarios is not to accurately represent interactions with virtual or physical representations but to identify fitting metaphors to assist in contrasting them toward building new theory. In both scenarios, an individual, or a group of individuals, encounter an interactive system. This individual or a group of individuals will henceforth be referred to as “users”, borrowing a term from user experience design.

### Virtual frame

First, consider a “virtual” representation, presented on, for example, the screen of a computer, a mobile device, or a large screen. In this frame of mind, users think that they are in a theater play where there is a script for interaction, and they may or may not have a role. Someone has set this experience up for them to watch or to take part in. Their role is narrowly and concretely defined. There are specific and limited norms within which the characters function. Users can participate if that is the role that is defined for them, as in a street and interactive theater. It is an experience, a drama, a comedy, a story. Users will learn; they will be moved; and they will be stimulated in this experience.

### Physical frame

Now, consider a “physical” representation, presented as a product in the user's environment. In this frame of mind, users ask the question, “what am I encountering,” and engage in broader sense-making by searching through a discrete set of familiar models of interaction with physical entities. They ask questions that are similar to what an individual in a hunter-gatherer society encountering a wild animal might ask, such as “what is it,” “what are its intentions,” “am I in danger,” “will it eat me,” “can I eat it,” “should I run away,” “should I get help to hunt it,” “can I use it to my benefit,” and so on. The answers to these questions might lead the individual to actions such as “hunting” the wild animal, using it as a “tool”, e.g., in farming, or training it to serve as a “collaborator” or “assistant” for security, hunting other animals, surveillance, and companionship.

Although the paper focuses on “virtual” representations presented on a two-dimensional screen and “physical”, three-dimensional representations presented in the shared space of the user to allow for contrast and analysis, the design space for interactive systems involves other possibilities for embodiment, including the blending of virtual and physical embodiments, mediation of physical embodiments by communication technologies, and immersive technologies that enable situated interactions with virtual embodiments. The paragraphs below briefly discuss these alternative possibilities.

### Blended frames

Although this paper compares and contrasts virtual and physical representations as distinct possibilities in the design of interactive systems, there are also ways in which these frames are “blended”, such as the receptionist robot designed by Gockley et al. (2005) and the instructional agent designed by Andrist et al. (2017). In these examples, the virtual representation is stripped from any context of its own, embedded in the physical environment of the user, and expected to act on this environment, such as making references to objects of interest for the user. Therefore, virtual representations can be integrated into the physical environment in a way that enables situated action.

### Mediated frames

Alternatively, physical embodiments can be experienced over media, such as a video stream of a remote environment where a robot is present, which removes the physical embodiment from the environment of the human observer and also removes the opportunity for situated action. Li (2015) observes that mediated physical embodiments are more similar to virtual embodiments than to physical embodiments in terms of interaction outcomes and thus highlights the role of physical presence in exploring the embodiment question. Similarly, Leyzberg et al. (2012) compare learning gains facilitated by a collocated robotic tutor against a video representation of the robot and demonstrate the positive effects of physical presence in interaction outcomes.

### Immersive frames

Virtual representations can also be presented in immersive environments, such when the user is immersed in the virtual agent's environment using virtual reality technology or the virtual agent is immersed in the user's environment using augmented reality technology. In these presentations, user experience closely resembles situated interactions with physical agents, as shown by Liu et al. (2017), although stereo vision cues are essential for immersive technologies to offer a similar experience to situated interaction with physical agents.

The remainder of this paper will focus particularly on distinctly virtual and physical representations such that the agents are situated either in virtual or physical environments. The next section of the paper will provide a more detailed analysis of these two frames of mind by contrasting the two frames in the context of a number of social, cognitive, or ecological mechanisms. These mechanisms, including “situativity, interactivity, agency, proxemics, and believability,” offer different lenses to understand the fundamental differences in human experience with virtual and physical representations for systems.

## Mechanisms

The mechanisms discussed below, “situativity, interactivity, agency, proxemics, and believability,” represent constructs where marked differences can be observed in human experience following encounters with virtual and physical representations. The discussion of each mechanism first provides a brief introduction to the mechanism and then highlights how the virtual and the physical frames differ with respect to the mechanism.

### Situativity

The first mechanism is the notion of “situativity”, the idea that human knowledge, thinking, and learning is situated in experience and environment (Greeno and Moore, 1993). The theory of situativity is an umbrella theory that encapsulates all “situated” psychological theories, including situated cognition, situated learning, ecological psychology, and distributed cognition. All of these theories to some extent go back to ecological psychology and the theory of affordances that suggest that properties of the environment, which Gibson, 1979 called “affordances”, elicit cognitive processes, including sense-making and learning. Other theories under the situativity umbrella such as distributed cognition further detailed this notion to describe cognition as a dynamic and social process.

In the situativist description of human experience, the individual is in the same environment with other agents, observing and experiencing the same things. The environment is social, dynamic, and evolving. The individual and the other agents can manipulate the same environment; they can collaborate; and they can change the real world. The social and situated nature of the activity also facilitates learning, so children learn the shared practices of their society by learning how to use artifacts and collaborate with others.

From a situativist point of view, how do people experience physical and virtual representations?

#### Physical frame

In the physical frame, the physical representation, e.g., the robot, is co-situated in and expected to act on affordances in the user's environment. In this frame, the environment is the shared physical environment, often constructed or shaped by the user—such as the user's home—and the robot can act on this environment. The user has chosen what is in this environment, shaped it in a way that would make life easier, and ascribed meaning to the artifacts in the environment. The robot comes into this environment as an independent agent, and the user expects to interact with the robot and the robot to act on the environment using the same affordances.

To illustrate human experience in the physical frame with a situativist lens, consider Minnie, a reading companion robot designed for middle schoolers Michaelis and Mutlu (2017, 2018, 2019). The robot is designed as a tabletop robot, and the envisioned interaction with the robot involved reading to the robot while sitting at a table with a book placed between the user and the robot. However, field observations of children using the robot showed that, despite this envisioned use, children integrated the robot into their environment in a way that best suited their use of the environment and supported their actions in that environment, as predicted by the theory of affordances. The robot was designed to be used in a particular way, but users appropriated it to fit into the day-to-day practices of how they used their environment. If they preferred to sit on a couch to read, they placed the robot on the couch or an ottoman and propped it up with a pillow. Similarly, if they prefer reading on the floor, they placed the robot and laid out the reading materials on the floor.

#### Virtual frame

In the virtual frame, the environment is a stage, as in a theater, constructed with a set of props that can be acted upon by different agents. The props are chosen to be consistent with the narrative. The user is aware that there is a greater plan that integrates the narrative, the characters' behaviors, and the environmental props. The user is usually familiar with the norms of the environment, and the affordances in the environment serve as opportunities for action for the user. Consider most video games, where characters are staged within well-crafted environments and affordances are explicitly delineated: a path that can be followed, a sword that can be picked up, a door that can be opened. Virtual representations can also be intentionally designed such that what the user can act on and what other agents in the environment can act on are not clear or consistent. The unscripted environment opens up the possibility of exploration, as affordances invite action as characterized by Gibson, 1979. Consider video games where players can seek hidden treasures, passages, or props to invite open engagement and exploration.

A physical agent and a virtual version of it do not offer vastly different possibilities and invitations for action, but when the representations are situated in their intended and eventual environments, these invitations diverge, highlighting the differing frames of mind in terms of situativity. In the physical frame, the unscripted environment opens up possibilities for exploration, and affordances invite action. The literature on human-robot interaction is rife with reports of children and adults engaging with robots in ways that are unexpected by the designers of these systems, such as bullying Keijsers and Bartneck (2018) and abusing Nomura et al. (2016) the robot as well personalizing Sauppé and Mutlu (2015) and attributing preferences Lee et al. (2012) to the robot. In these interactions, people explore possibilities and push boundaries with robots just like they explore possibilities in human interactions and in nature. When the environment is prescribed, as it is in the virtual frame, action must be directed by scripted behavior, as the user cannot openly explore. In this sense, while virtual environments appear as if everything is possible, all these possibilities require prescription, so nothing is possible unless it is explicitly created. In the physical world, these possibilities are cued and explored by people.

To summarize the situativist perspective, a virtual environment is designed to immerse the user in the environment of the agent, while a robot is designed to be successfully immersed in the human environment.

### Interactivity

The second mechanism is “interactivity”, particularly how human interactions with virtual and physical representations are choreographed and how this interaction unfolds. What is the structure of interaction? What are its elements? How can we make sense of this multifaceted and complex concept? Here, “theater” provides a more appropriate context to study the structure of interaction than psychology does, given its focus on “synthesis” instead of “analysis”.

What is a theater? The origins of a theater go back to Greeks in the sixth century BC, particularly to the festivals held in Greek cities where historical or recent events were depicted for the citizens Hartnoll and Brater (1985). These depictions then further diverged into different forms, from comedy to tragedy, including interactive theater, during the Greek period. What is the structure of a theater piece? Aristotle, as described by Butcher, 1902, offered a model of tragedy that includes six elements: (1) *mythos*: plot/narrative; (2) *ethos*: character; (3) *dianoia*: thought; (4) *lexis*: diction; (5) *melos*: melody; and (6) *opsis*: spectacle. In this model, earlier elements represent abstract plans, goals, and structure of the play, and later elements denote concrete actions, dialog, and so on.

Relevant to the interactivity lens is the plot or the narrative, which follows a particular structure, an “archplot”, in all storytelling arts, including theater, film, and fiction. In this structure, also known as “Freytag's pyramid” Freytag and MacEwan (1908), the plot is opened with an “exposition”, followed by a “rising action”, followed by a “climax”, followed by a “falling action”, and eventually a “resolution”, or “dénouement”. From Shakespeare's Hamlet to Mario Puzo's Godfather, most plots can be mapped onto this structure. Even in an interactive theater, the plot is designed such that there are key points at which the audience is brought into the plot and asked to participate in the narrative. This participation could result in alternative climaxes or resolutions, but there is still a clear exposition and finale.

In virtual environments, such as virtual reality, this archetypal structure has been recognized as a problem because the scripted nature of interactions in these environments and the freedom that the environment affords for movement and exploration presents a conflict. More formally, this problem, called by Aylett (2000) the “narrative paradox”, represents the conflict between pre-authored narrative structures, especially the plot, and the freedom a virtual environment offers a user for physical movement and interaction. Virtual environments exemplify “immersive” frames: users are invited into the prescribed environment of virtual characters, while they also have the ability to move and explore the environment. To address this paradox, researchers have proposed “emergent” narrative structures inspired by interactive theater that involve dramatic episodes that invite audience participation and facilitate affective involvement Aylett et al. (2005).

Dramatic structure can also be applied to everyday, real-life interactions. The idea of “whole actions” from drama, where the beginning, middle, and end of an interaction has a “dramatically pleasing shape” Laurel (2013), is similar to the “rituals”, patterned ways in which people start, end, and manage interactions, such as greetings, farewells, and affirmations, that people follow in social interaction Goffman (1967). Where drama and real-life interaction diverge is the “emergent” process in which people coordinate actions and communicate in order to accomplish a shared goal that may or may not be known to individuals at the onset of the interaction. To facilitate this emergent process between humans and robots, research in robotics has drawn on theories of “joint action” and “joint intention”. Joint action can be characterized as any form of social interaction whereby two or more individuals coordinate their actions in space and time to bring about a change in the environment Sebanz et al. (2006). Joint intention theory highlights the importance of commitment to a shared goal and communication to ground mutual beliefs in teamwork. Building on these principles, this research has developed physical agents that can align their goals with those of their users and coordinate their actions to accomplish them Alami et al. (2006); Mutlu et al. (2013).

#### Physical frame

In the physical frame, the narrative of the interaction emerges from joint action and intention between the user and the robot. Consider an interactive theater where audiences are brought into a scripted play and are participating in the unfolding of a predetermined story. For example, Laurel (2013) describes an interactive children's play where Alan-a-Dale, the minstrel from Robin Hood, bringing the audience into the story by leading them around a park where the play is performed. In contrast, in this frame, the robot is brought into and is participating in the emergent narrative of the user's life. Examples include a robot that is designed by Tamura et al. (2017) to listen to children's stories and a robotic assistant designed to proactively offer help to its user whenever it can Baraglia et al. (2017).

#### Virtual frame

In the virtual frame, similar to an interactive theater, the user participates in a narrative crafted for engagement in the virtual environment. To improve user engagement and break monotony, research into virtual representations have explored ways of enabling users to participate in the shaping of the narrative plot, for example, by inviting the user to actively select behaviors for characters in the virtual environment (e.g., Aylett et al. (2005)) and by monitoring user physiology and adapting narrative structure to measured physiological responses, enabling the user to passively shape the narrative structure (e.g., Baraglia et al. (2017)).

Although physical robots have also been used to create well-crafted interactive experiences, such as in theater plays Breazeal et al. (2003); Zeglin et al. (2014), theme parks Cornfeld (2019), and museums Yamazaki et al. (2010), these experiences differ from day-to-day human-robot interactions involving robotic products and more closely resemble interactions with virtual characters with respect to the interactivity mechanism.

To summarize the interactivity mechanism, in the physical frame, the narrative of the interaction emerges from joint action and intention between the user and the robot, while the virtual frame involves the user participating in a narrative crafted for engagement in the virtual environment.

### Agency

The next mechanism is the notion of “agency”. What is agency? How does it manifest itself in interactions with virtual and physical representations? The notion of agency was again defined first by Aristotle who was trying to make sense of how things come about, what makes them, what materials were used, and so on. Aristotle characterized four “causes,” including the “agent”, or the “efficient cause,” describing it as what causes an object to change, move, or rest Falcon (2019). In his text, “Physics,” Aristotle Reeve (2018) gives the example of a sculptor building a sculpture, and the agent here is the sculptor who is bringing change to a piece of stone.

More recent perspectives connect agency to the theory of affordances; Withagen et al. (2012) argue that affordances in the environment not only serve as action possibilities but also “invite” agents to take action. In that sense, affordances are not impartial features of the environment, but they actively encourage agency. Humans and animals are not agents programmed by their environments, but they instead choose what affordances to act on based on the value and meaning of the actions that they represent Reed (1996). Therefore, agent behavior results from an interplay between the solicitations of the environment for action through affordances and the agent's choices and capabilities to navigate these possibilities based on its goals and values Withagen et al. (2017).

More than a decade ago, Mutlu and Forlizzi (2008) conducted an ethnography of one of the very few robots that had been introduced into day-to-day human environments. The robot, called Tug, made deliveries at a hospital, such as transporting blood samples from a nurse's unit to the lab. In making deliveries, the robot autonomously navigated in the environment, opened doors, controlled elevators, and so on. As an autonomous robot, it exerted a high level of agency in the environment. It used the affordances of the environment to take action, guided by its goals and intentions. Consider the following quote from one of the users of the robot:

[The robot] doesn't have the manners that we teach our children, and it takes precedence over people most of the time … I sort of find it insulting that I stand out of the way for patients or a gurney or a wheelchair coming through, but [the robot]—just barrels right on … You need get out of the way [for the robot].

Here, a hospital worker is complaining that the robot lacks the manners that would be expected from a worker. It used the environment—the hallways, the elevators—to achieve its goals with disregard to the goals of other people in the environment. Consider another quote:

I called them nasty names and told them, “Would you shut the hell up? Can't you see I'm on the phone? I'll get to you. If you say, ‘[the robot] has arrived,’ one more time, I'm about to kick you in your camera.”

Here, another worker is complaining that the robot would arrive to make a delivery and make announcements until someone picked up the delivery. Although hospitals are early adopters of cutting-edge technology, the robot is the only form of technology that had the level of agency to come to users and assert itself, interrupting users during their work. The use of all other technology was at the discretion of the users. To use a mobile patient monitor, users walk up to the device, carry it around, and press its buttons to interact with it. The robot, on the other hand, acts as an independent agent with its own goals that often came into conflict with the goals of its users.

#### Physical frame

In the physical frame, the robot is seen as high agency, pursuing its own meaning as an independent agent, as a result of its autonomous, unbounded, and thus less predictable, behavior. Robots designed for delivery, hospitality, escort, or surveillance applications in public spaces elicit particularly strong perceptions of agency by passersby who are unaware of or unfamiliar with the robot's purpose, goals, or state.

#### Virtual frame

In the virtual frame, engagement is at the discretion of the user due to lack of physical autonomy and to the scripted, bounded, and thus more predictable behavior. Users choose to initiate, maintain, and end interaction with the virtual representation.

To summarize, in the physical frame, the robot is an independent agent using the affordances of the human environment to pursue its own goals. The virtual frame, on the other hand, gives discretion to the user, relinquishing part of the agency of the virtual representation and requiring users to initiate interaction.

### Proxemics

The fourth mechanism is “proxemics”, which in a sense is a consequence of the “situativity” mechanism. When artifacts of media and technology are placed in the human environment, they necessarily enter the realm of spatial relationships among bodies. Human perceptions of space, particularly of spatial relationships among agents, are shaped by culture, follow culture-specific patterns, and are internalized as norms that are applied unconsciously and consistently by members of the culture Hall (1963).

Proxemic interactions between humans and characters in virtual reality and between humans and robots have been studied extensively. The first quote from Mutlu and Forlizzi (2008) provided in the previous mechanism highlights the importance of considering these interactions, particularly the negative effects of any violations of these norms. The hospital delivery robot lacked the ability to recognize or follow these norms and used its environment in a way that optimized for its navigation, eventually frustrating the cohabitants of the environment. The quote highlights that the robot lacks “proper manners” for proxemic interaction.

The social psychological literature has proposed that proxemic interactions are governed by the need for individuals to regulate intimacy; specifically, Argyle and Dean (1965) suggested that interaction partners establish an “equilibrium” for interpersonal intimacy using a number of social cues including gaze, physical proximity, intimacy of the conversational content, and amount of smiling, which the authors expressed using Equation 1.

(Equation 1) $$Intimacy=f\left\{ \begin{array}{l} \text{eye contact}\\ \text{physical proximity}\\ \text{intimacy of topic}\\ \text{amount of smiling}\\ \text{etc}. \end{array}\right.$$

According to this model, in human interactions, individuals will seek to maintain this equilibrium with their interlocutors and thus will match attempts to increase intimacy by others, e.g., an increase in eye contact, with a reduction in other behaviors, e.g., by moving away from them. Bailenson et al. (2001) tested how well this model predicted proxemic behaviors between humans and characters in virtual reality. In the study, participants were asked to approach virtual character to retrieve a number placed on the back of the character, and the study manipulated the human likeness of the character and whether the character maintained eye contact with the participant. The results showed that participants maintained a greater distance from the character when the character was humanlike than when it was not and when the character maintained eye contact than when it did not, although the latter effect was only present among females. As predicted by the model, these participants changed their distance in an attempt to regulate their intimacy with the virtual character, but not the non-character object, and when the character attempted to increase intimacy by establishing mutual gaze.

Kaplan et al., 1983 proposed that this distancing behavior would be moderated by likability between the interaction partners and suggested four competing models for how likability and distancing would interact. Mumm and Mutlu (2011) tested these models to better understand proxemic behavior between humans and robots using the same task used by Bailenson et al. (2001). Their results showed that human-robot proxemic relationships were most consistent with the “attraction-transformation” model, that is, when partners attempt to increase intimacy by increasing eye contact, people attempt to regulate their intimacy with unlikable partners by distancing themselves from them, but not with likable partners. These studies highlight the prominence of proxemics for human-machine interactions, particularly the human ability, and the need to use proxemic behaviors to manage interactions with others.

But what are the proxemics of virtual representations? Although proxemic relationships between human and virtual characters depend, to some extent, on how the character is presented to the user, for virtual representations that appear on a screen, the spatiotemporal relationships are similar to those between performers and the audience in a theater. Consider plays involving music performed by an orchestra who would usually be seated in the orchestra pit. Composer Richard Wagner called the gap between audience and actors created by the orchestra pit the “mystic gulf,” which placed strong constraints on the proxemic relationship between actors and spectators Kennedy (2018). Unlike physical representations that are situated in the human environment and has the ability to engage in a wide range of proxemic behaviors with users, such as freely entering into and exiting from the user's personal space or approaching the user from behind, interactions with virtual representations involve a largely fixed proxemic arrangement in orientation and distance, primarily vis-à-vis at a reading distance, due to the screen-based presentation of the virtual representation.

#### Physical frame

In the physical frame, proxemics is dynamic, co-managed, and consistent with human proxemic norms. Physical representations have the ability to display proxemic behaviors and enter into different spatial relationships with their users, and their users can respond to these behaviors and manage these spatial relationships. Because humans expect these relationships to follow culturally situated patterns, proxemic behaviors by physical representations can easily violate human expectations. In the physical frame, users enter into a finely coordinated, well-choreographed dance with machines, and their experience is shaped by their existing relationship with the machine and its ability to appropriately distance itself from them.

#### Virtual frame

In the virtual frame, proxemics is constrained, determined by conventions and physical arrangements. Across various presentations, including on computer screens, mobile devices, or large displays, the proxemic arrangement involves a “mystic gulf” between the virtual representation and its user due to the physical-virtual divide and the vis-à-vis orientation that is necessitated by the physical characteristics of the technology.

Li (2015) argued that physical collocated presence is the main differentiating factor between virtual and physical embodiments. It is possible that collocated presence provides opportunities for exploring and regulating different proxemic relationships, while the relatively fixed and constrained proxemic arrangements with virtual representations limit such exploration. Additionally, immersive frames can eliminate this gulf between the user and virtual representations and can similarly provide opportunities for exploring different proxemic relationships.

In summary, in the physical frame, proxemics is highly dynamic, co-managed by all agents involved, and consistent with human proxemic patterns and norms, while the proxemic norms in the virtual frame are highly constrained and determined by specific conventions and physical arrangements.

### Believability

The next and last mechanism is “believability”. To interact with artificial agents, to engage with them at an interpersonal level, and to experience internal states such as emotion and motivation, users must possess a level of “belief” about the agent and a willingness to put aside the conscious awareness that the agent is only a representation. This notion is often referred to as “the willing suspension of disbelief,” coined by Taylor Coleridge in the 19^*th*^ century about how readers can appreciate and enjoy poetry—by intentionally believing that it is real for the moment—which he also called “poetic faith” Ferri (2007). This idea has been applied to novels, theater, film, advertising, and interactive games.

The last half-century has seen debate among philosophers about how individuals experiencing fictional characters and events can suspend disbelief about the fictional nature of the material but not be able to suppress strong emotion—a concept they called the “paradox of fiction” Radford and Weston, 1975; Radford and Weston (1975); Walton (1978). More generally, psychologists refer to the uncomfortable state in which individuals hold conflicting beliefs, ideas, or values as “cognitive dissonance” Festinger (1957). When people hold “dissonant” ideas, they change their behavior or create with ways of thinking or rationalizations that restore a state of “consonance”. In the case of managing the paradox of fiction, individuals might be engaged in beliefs of different orders, such that they hold a “first-order belief” that they are experiencing fiction, and given that belief, they can hold “second-order beliefs” about fictional characters and events. Thus, having second-order beliefs in and emotional reactions to fiction requires first-order beliefs about fiction Schaper (1978).

More recently, researchers have argued that emotional responses to fiction may be modulated by whether or not the content is relevant to the viewer. Using physiological measurements, Sperduti et al. (2016) found that people have similar emotional reactions toward real and fictional stimuli that are relevant to themselves, but fictional stimuli loses its emotional effect over real stimuli when they are not relevant. For example, consider the context of video gaming; given the first-order belief that users are playing a video game, they are willing to hold the second-order belief that they are fighting a fierce creature and fully experience the emotions that are associated with defeat and victory. In this example, fictional stimuli that is not relevant, as users know that they will not be attacked by a fierce griffin in real life, provides a safe environment to hold second-order beliefs and experience emotion.

How does the suspension of disbelief work in interactions with physical representations? Consider the following quote from the study of the hospital delivery robot by Mutlu and Forlizzi (2008):

Well, it almost ran me over … I wasn't scared … I was just mad … I've already been clipped by it. It does hurt.

Physical representations, given their high agency and physical autonomy, might short circuit people's assessments of whether or not the robot is relevant to themselves. Namely, the robot makes itself relevant by inserting itself into the user's personal space and, often inadvertently, seeking attention. In the quote above, the user indicates being harassed by the robot and experiencing physical harm, which forcibly takes the experience from being non-relevant to being self-relevant and potentially from fictional to real. The high level of agency, discretion on the side of the robot to engage in interaction, and potential for physical harm provide people with a first-order reality that they have to somehow interact with the robot. Duffy and Zawieska (2012) provide a more extensive discussion of suspension of disbelief with robots.

#### Physical frame

In the physical frame, agency and physical autonomy establish self-relevance and bring fiction into reality. Physical representations are perceived as being more self-relevant due their agency in the user's environment, which shifts the first-order belief that one is experiencing fiction to a sense that one has no choice but to deal with a real agent.

#### Virtual frame

In this frame, the representation offers a safe environment to experience emotion. This frame involves the first-order belief that one is experiencing fiction, and given this belief, allows one to engage with characters and events emotionally and even engage in situations that would be too emotionally difficult to experience in real life, such as fighting a griffin or experiencing the deathbed conversation of an old couple. This ability to support an emotional experience in the safety of first-order assumptions has inspired clinical and other applications such as a virtual reality experience where people can practice self-compassion by interacting with a depressed child Falconer et al. (2016).

Consider the scenario, provided earlier in the paper, involving the individual who lived in a hunter-gatherer society that encountered a wild animal. In the physical frame, the wild animal is seen as being self-relevant to the extent that it can physically affect, or even physically harm, the individual, as illustrated by the quote from Mutlu and Forlizzi (2008)—“it does hurt.” In the virtual frame, such as in a video game where the individual might encounter unfamiliar characters and ask similar questions, the individual has the first-order belief that the stimuli is not self-relevant and thus cannot harm the individual, and thus, the individual allows oneself to experience emotion.

In summary, the virtual frame involves the first-order belief that one is in a fictional world, e.g., “I am in a theater play,” where they allow themselves to experience emotion and relate to characters, while the physical frame involves sense-making about the intentions and actions of an agent that exerts its agency on its environment and people in it, e.g., “What am I facing?”

## Discussion

This paper aimed to offer a provisional answer to the “why” of the “embodiment question”. There is mounting evidence that “embodiment”—the state of possessing a discernible body—and the form that this embodiment takes, from abstract graphical representations to highly realistic surrogates, significantly affect human experience with artificial agents. Specifically, the literature predicts that physical embodiments will elicit stronger interaction outcomes than virtual embodiments Deng et al., 2019. This paper argued that these forms of embodiment not only differ in the magnitude of the responses they receive from people but also elicit fundamentally different frames of mind in their users. Rather than providing a conclusive explanation, it aimed to characterize a set of provisional mechanisms that can serve as blueprints and testable hypotheses for future research. The paragraphs below provide recommendations for research and design and revisit the case study described in §1 to contextualize the presented mechanisms.

### Implications for research

The majority of research on embodiment asks the question “which embodiment is better,” such as the 63 articles included in a review article by Deng et al., 2019 that compared a physical embodiment to one or more other forms of embodiment. Although these studies have been informative, there is a greater need for studies that explore how mechanisms work across different representations to understand the types of interactions they are best suited to support. This research should involve study designs and scenarios that allow systematically studying different facets of key mechanisms, such as the set of mechanisms presented in this paper, rather than mere comparisons of representation. For example, to investigate situativity, studies might compare how a virtual agent making references to objects in the virtual environment might differ from one that makes references to objects in the physical environment. These studies should also adopt methods, such as the system-level evaluation proposed by Peltason et al. (2012) and the multivariate evaluation method proposed by Huang and Mutlu (2014), that enable the study of a large number of parameters of a system, as studying complex mechanisms by isolating facets may not be feasible.

### Implications for design

What are the implications of this analysis for the design of machines for human interaction? An extension of the proposal that virtual and physical representations elicit fundamentally different frames of mind is the argument that “certain representations are better suited to support certain tasks and applications” given their different characteristics with respect to the mechanisms discussed in this paper. Of note is that, in practice, different representations are chosen for different applications primarily due to technological or practical considerations, and the argument here is that “experiential” differences that are elicited by the mechanisms discussed above between these representations should also motivate such differential use. In brief terms, certain representations are better suited to support certain tasks and applications.

To summarize the characteristics of each representation, in the physical frame, representations are co-situated in the user's environment; the interactions emerge through joint action and intention; they are seen as independent agents pursuing their own goals; proxemic relationships with these agents are dynamic and co-managed to follow human norms; and the agents are seen as real-world, self-relevant stimuli. In the virtual frame, on the other hand, the user is brought into the agent's environment; they are invited to participate in a crafted narrative; engagement with the representation is at the user's discretion; proxemic relationships are constrained and determined by physical arrangements and conventions; and the virtual environment serves as a safe setting to experience emotion. Table 2 summarizes the characteristics of the two representations across each mechanism, which, as more evidence is obtained to support them, can directly guide design.

**Table 2:** Summary of the characteristics of physical and virtual frames with respect to the mechanisms reviewed in this paper

Mechanism	Physical	Virtual
Situativity	Co-situated in the user's environment	User is brought into the agent's environment
Interactivity	Emerges from joint action/intention	Invites users to participate in a crafted, patterned plot
Agency	Seen as independent agent pursuing own goals	Engagement is at the user's discretion
Proxemics	Dynamic, co-managed to follow human norms	Constrained, involving learned conventions
Believability	Real-world, self-relevant agent	Safe environment to experience emotion

These conclusions lead to a number of clear design recommendations. For example, the “physical frame” best supports representations that can act as physical, situated collaborators and assistants engaged in activities that are interspersed across time and space and situated in day-to-day life. The “virtual frame”, on the other hand, best supports representations that act as counselors, instructors, and coaches that engage in focused, time-bound activities through interactions that are metaphorical, rich, and crafted. Table 3 summarizes the applications, activities, and types of interactions that can be afforded by representations that elicit each frame.

**Table 3:** Applications, activities, and types of interactions that can be best supported by physical and virtual representations

Characteristics	Physical	Virtual
Applications	Physical, situated collaboration, assistance	Counseling, instruction, education, coaching
Activities	Activities interspersed across time and space	Focused, time-bound activities
Interactions	Interactions situated in day-to-day life	Metaphorical, rich, crafted interactions

Prior research in human-robot interaction has also argued that designers must seek an appropriate “match” between a robot's task and its appearance and behavior, such that the capabilities of the robot are justified by the requirements of the task Goetz et al. (2003). Studies that compared virtual and physical embodiments across different tasks concluded that these embodiments were better suited to support different types of tasks Hoffmann and Krämer (2013): virtual embodiments are perceived as being more competent in conversational tasks, while physical embodiments are seen as being more competent in guiding users through a physical task situated in the real world.

The recommendations offered above do not imply that systems with physical representations will always be more effective collaborators or that systems with virtual representations will always be more effective tutors, as these outcomes will be affected by a number of factors, including the specific design of the system, the characteristics of the task at hand, the preferences and orientation of the user, and so on. These recommendations aim to offer a starting point and assist designers in considering trade-offs and making informed representational choices that must be validated and refined through iterative design.

These recommendations should also be evaluated holistically and in the context of the analysis provided above. For example, although Table 3 suggests that instruction applications might be best supported by virtual representations, the reader should also consider the characteristics of the instructional activity and the basis of this recommendation with respect to the mechanisms discussed above. Therefore, instruction that is situated in the user's environment and interspersed across time and space might be best supported by physical representations, as found by Hoffmann and Krämer (2013), or by augmented reality technologies that embed virtual characters into the user's physical environment. From a situativist point of view, both solutions would support the need to situate the instructional activity in the user's environment in order to draw on its affordances.

### Case study revisited

To motivate the analysis presented earlier, this paper used a case study where different subsets of hypothesized effects were observed across virtual and physical representations. What are the implications of the analysis provided here and the summary argument made above for the findings of this case study? Do they offer plausible explanations for these observed differences? As summarized in Table 1, the study found that gaze supported conversational mechanisms as expected for both the agent and the robot, but the agent with cognitive gaze aversions was not seen as thoughtful, and people did not disclose more to the robot using intimacy-regulating gaze cues.

The characterization made earlier that “virtual representations” are crafted experience in which to safely feel emotion and physical representations are independent agents pursuing their own goals offers tentative explanations to these findings (summarized in Table 4). For example, if the robot is a seen as an independent agent with its own goals, one may not disclose information to an independent agent with whom one has not yet established trust, and thus, participants did not disclose more to the robot when it displayed appropriate gaze behavior. Similarly, one might make attributions of mind to an independent, co-situated agent, e.g., the robot, but not to a virtual representation that is seen as crafted experience by an author who may be the target of such attributions, and thus, the virtual agent was not seen as being thoughtful when it displayed appropriate gaze behavior. Finally, if one feels safe to experience emotion, disclosure might naturally follow, which is what was observed with the virtual agent.

**Table 4:** Mapping characterizations of virtual and physical frames to the findings of the case study

	Virtual	Physical
	Crafted experience to safely feel emotion	Independent agent with own goals
	↓	↓

Task	Agent	Robot
Attributions of thoughtfulness	Not supported	Supported
Disclosure	Supported	Not supported

These explanations do not aim to suggest that virtual representations will not be seen as being thoughtful or that physical representations will not support intimacy. Participants may have attributed thoughtfulness not to the agent but to the author, creator, or programmer of the virtual character, and disclosure to a robot might take time until trust between the robot and its user has been established. These are tentative explanations that must be tested, thought the ability to derive such explanations illustrates the promise of the analysis provided in this paper to guide future research and design.

### Limitations of the study

The application of the main hypothesis of this paper and the mechanisms discussed to the case study comes with a number of limitations. First, the differences between the virtual and physical embodiments used in the two studies might serve as an alternative explanation for the differences in participant disclosure. The stylized humanlike appearance of the virtual agent might have facilitated disclosure but not the abstract, product-like appearance of the physical robot. Additionally, in the study, the robot signaled its gaze shifts by moving its head, while the virtual agent used a combined eye and head movements following a biomechanical model of human gaze Andrist et al. (2012). Zhang et al. (2017) argued that subtle gaze movements are essential to achieve social outcomes such as intimacy regulation, although more research is needed to demonstrate that head movements are insufficient to achieve such outcomes. Furthermore, the design of the virtual agent included a humanlike mouth that displayed social smile at all times, while the robot lacked an articulated mouth. Thus, unlike the agent, the robot might have been seen by participants as lacking the social cues necessary for “empathy”, which might be essential for disclosure. Paiva et al. (2017) argue that “empathic responses” that signal the agent's internal states using verbal and nonverbal cues facilitate perceptions of agents as empathic characters.

Second, the studies included in the case study involved brief interactions with the virtual agent and the robot, and the studied outcomes might change over long-term interactions. For example, although participants might have seen the robot as an independent agent with its own goals and thus not have disclosed more information when it employed intimacy-regulating gaze cues, they may do so after repeated interactions help build rapport and trust. Third, the goal of the discussion of the applications, activities, and types of interactions that may be best supported by virtual and physical representations provided above is not to make definitive statements but to offer provisional guidelines that must be tested by future research or through iterative design.
